# Epigenetics, ethics, law and society: A multidisciplinary review of descriptive, instrumental, dialectical and reflexive analyses

**DOI:** 10.1177/0306312719866007

**Published:** 2019-08-01

**Authors:** Charles Dupras, Katie Michelle Saulnier, Yann Joly

**Affiliations:** Centre of Genomics and Policy, McGill University and Génome Québec Innovation Centre, Canada; Centre of Genomics and Policy, McGill University and Génome Québec Innovation Centre, Canada; Centre of Genomics and Policy, McGill University and Génome Québec Innovation Centre, Canada

**Keywords:** biologization, discrimination, ELSI, epigenetics, knowledge translation, nature versus nurture, privacy, reproduction, responsibility and justice

## Abstract

Epigenetics, defined as ‘the study of mitotically and/or meiotically heritable changes in gene function that cannot be explained by changes in DNA sequence’, has emerged as a promissory yet controversial field of scientific inquiry over the past decade. Scholars from many disciplines have formulated both optimistic and cautionary claims regarding its potential normative implications. This article provides a comprehensive review of the nascent literature at the crossroads of epigenetics, ethics, law and society. It describes nine emerging areas of discussion, relating to (1) the impact of epigenetics on the nature versus nurture dualism, (2) the potential resulting biologization of the social, (3) the meaning of epigenetics for public health, its potential influence on (4) reproduction and parenting, (5) political theory and (6) legal proceedings, and concerns regarding (7) stigmatization and discrimination, (8) privacy protection and (9) knowledge translation. While there is some degree of similarity between the nature and content of these areas and the abundant literature on ethical, legal and social issues in genetics, the potential implications of epigenetics ought not be conflated with the latter. Critical studies on epigenetics are emerging within a separate space of bioethical and biopolitical investigations and claims, with scholars from various epistemological standpoints utilizing distinct yet complementary analytical approaches.

## Introduction

The term ‘epigenetics’ was used by Conrad H. Waddington from 1942 to designate the ‘[causal] mechanisms by which the genes of the genotype bring about phenotypic effects’ (Waddington, 1942: 18). In the early 1990s, Waddington’s concept gradually changed to reflect a rise in investigations at the molecular level, which aimed to better understand variations in gene expression, some of which were relatively stable in time and thought to be influenced by the environment (Jablonka and Lamb, 2002). Today, epigenetics is defined as ‘the study of mitotically and/or meiotically heritable changes in gene function that cannot be explained by changes in DNA sequence’ (Russo et al., 1996: 1). It has developed into an increasingly diversified field of scientific inquiry, with researchers focusing on, for instance, associations between toxic exposures and epigenetic modifications (e.g. DNA methylation, histone modifications), epigenetic variants and diseases, or the possible inter-/transgenerational inheritance of acquired epigenetic traits.

Scientific advances in epigenetics have recently captured the attention of a variety of researchers and stakeholders. Interpretations have been formulated regarding the field’s significance and possible impact on society at multiple levels, including medical (Balasubramanian, 2017; Rutherford, 2015), philosophical (Hens and Van Goidsenhoven, 2017; Spears, 2017), judicial (Hesman Saey, 2016), political (Goldberg, 2017; Smith, 2013) and commercial (GWG Holdings, 2018). Exciting subfields such as environmental or social epigenetics have been called ‘revolutionary’, because they could provide additional grounds to revisit some gene-centric theories, long perceived by many as too simplistic and reductionist of human identity, behavior and health. In this article, we present the results of a comprehensive literature review of potential epistemological and normative implications of epigenetics, as anticipated by researchers in the social sciences and humanities over the past dozen years.

We characterize nine ‘areas of discussion’ at the crossroads of epigenetics, ethics, law and society. We highlight the coexistence of optimistic and cautionary appraisals of the potential consequences of epigenetic research, and of its foreseen translations into health interventions and public policies. We also underscore the coexistence of four distinct yet complementary analytical approaches – descriptive, instrumental, dialectical and reflexive – employed by different authors from various disciplines. We then argue that while studies of epigenetics are producing apparent conflictual bioethical and biopolitical claims, the overall result is the production of an increasingly nuanced and sophisticated corpus. We conclude with a call for attention to additional burgeoning areas of discussion in the coming years.

## Method

### Search for publications

Two investigators (CD and KMS) independently searched Google Scholar for publications that address (potential) ethical, legal and social implications (ELSI) of epigenetics. Initial searches began in September 2016 using variations and permutations of keywords such as ‘epigenetics’, ‘DNA methylation’, ‘histone’, ‘ethics’, ‘law’ and ‘society’. The two investigators also identified articles using the snowballing method, by (a) iteratively scrutinizing the references of selected papers for missing publications, and (b) continuously searching for missing publications using the ‘cited by’ function in Google Scholar, in conjunction with some of our most frequently cited entries (>10 citations). The search ended in January 2018, when both investigators agreed that saturation had been reached.

### Inclusion criteria

We included refereed articles, commentaries and editorials published in English academic journals between 2000 and 2017 (inclusively). We considered the publication of two seminal scientific articles, one in the field of ‘nutritional epigenetics’ (Waterland and Jirtle, 2003) and the other in ‘social epigenetics’ (Weaver et al., 2004), to be at the origins of recent discussions regarding the ELSI of epigenetics. Hence, we could predict with a reasonable degree of confidence that expanding our search before the year 2000 would not generate additional relevant publications. To be selected, a publication had to describe and/or discuss one or more potential issues or implications related to the field of epigenetics. To account for the diversity of issues and implications explored in the literature, we included publications that touched on any ELSI in epigenetics, including broader implications of the field – such as epistemological (e.g. gene-centric views such as genetic essentialism and determinism) and methodological (e.g. for social sciences and humanities, or political theory) implications – that may be more descriptive and to some extent less normative/prescriptive.

### Exclusion criteria

We excluded publications whose content was mainly technoscientific or medical in nature (e.g. focused on epigenetic mechanisms), and in which the authors only superficially touched on the potential ELSI in epigenetics. Editorials or introductory remarks were excluded when they were solely descriptions of the content of an article in a special issue. Books, book chapters, governmental reports and theses addressing the topics were excluded. Our final list of selected publications included 99 peer-reviewed articles and 19 commentaries or editorials (see online Appendix).

### Analysis

All selected publications (*n* = 118) were independently categorized by two investigators (CD and KMS) for journal type (research discipline targeted), area of discussion, general tone (neutral, optimistic or cautionary) and analytical approach (descriptive, instrumental, dialectical or reflexive). We also flagged analyses based on the results of empirical studies. The two investigators’ tables were subsequently compared and merged. We sought to achieve consensus for every publication. In cases of first stance disagreement, we openly discussed our individual analyses and reasoning, and simultaneously scrutinized the papers again to reach consensus.

### Areas of discussion

After reading all papers selected by July 2017 (*n* = 78) – that is, six months before the search was ended – the two investigators agreed on nine general areas of discussion, relating to:

- the traditional *nature–nurture* dichotomy;
- the embodiment or ‘*biologization*’ of the social;
- *public health* and other preventive strategies;
- *reproduction*, parenting and the family;
- *political theory* (e.g. conceptual analyses of responsibility and justice theories);
- *legal proceedings* (e.g. implications for ‘tort law’);
- the risk of stigmatization, *discrimination* or eugenics;
- *privacy protection*; and
- *knowledge translation* (including discourse analyses).

Other discussions also emerged relating to, for instance, the appropriate definition of the word ‘epigenetics’ (Greally, 2018; Thayer and Non, 2015), the various origins of the field (Haig, 2004; Jablonka and Lamb, 2002) and the amount of evidence that has been accumulated so far on transgenerational epigenetic inheritance in humans (Heard and Martienssen, 2014; Whitelaw, 2015). Although important, these scientific debates were not considered areas of discussion in this review, as our intention was to focus on the potential ELSI of epigenetics. We also found important overlap between these debates and most of our areas of discussion.

### General tone

All selected publications were categorized according to the general attitude of the author(s) towards potential ELSI of epigenetics; that is, whether they expressed largely neutral, optimistic or cautionary views. A publication was considered *neutral* when the authors did not take a clear position; that is, when they neither ‘promoted’ nor ‘warned against’ a specific implication of epigenetics. A publication was considered *optimistic* when the authors discussed some aspect of epigenetics as representing an ‘opportunity’; that is, when they were ‘enthusiastic’ about its potential implications, and the overall focus of the paper was on the potential ‘benefits’ of this new field of study. A publication was considered *cautionary* when the authors discussed some aspects of epigenetics as potential ‘threats’; that is, when they were ‘concerned’ about its potential implications, and the overall focus of the paper was on the potential ‘risks’ of this new field of study. When publications contained both optimistic and cautionary elements, we categorized the publication based on overall tone of the paper – although defining the threshold for when a paper becomes naïvely enthusiastic or disproportionally alarmist was not the goal of this review. It is also important to note that we grant no superior prima facie value to any of the neutral, optimistic or cautionary analyses reported in this paper.

### Analytical approach

All selected publications were also categorized according to the main analytical approach adopted by the author(s): descriptive, instrumental, dialectical or reflexive. A publication was considered *descriptive* when the authors reviewed a set of potential implications or issues but remained distant about them. A publication was considered *instrumental* when the author(s) presented emerging findings about epigenetic mechanisms as evidence to persuade or convince the reader about an already existing claim (e.g. favoring more interdisciplinary work or allocating more financial resources to preventive public health initiatives). A publication was considered *dialectical* when the authors identified juxtaposed/contradictory arguments, depictions of epigenetic mechanisms or implications. This argumentative approach, defined by focusing on the multiple parts of an object or issue, considers these as results (synthesis) of underlying forces in tension (thesis, antithesis) that may sometimes be imperceptible at first glance. A publication was considered *reflexive* when the authors engaged in an ‘analysis of the analyses’ of the implications of epigenetics; that is, they considered the context of the knowledge, the knowledge users, and how epigenetics and the ELSI of epigenetics may be interpreted in certain circumstances. Finally, when publications contained more than one type of analytical approach, we only reported the most salient.

## Results

Over the past dozen years, discussions regarding the ELSI of epigenetics have emerged from an impressively large diversity of disciplines. This may be explained by the fields of environmental and social epigenetics standing at the intersection of human health and broader societal concerns. Sociologists and Science and Technology Studies scholars have been the most prolific so far, with an increasing number of bioethicists and legal scholars recently contributing. Although a few foundational papers were published between 2006 and 2012, starting in 2013, interest in epigenetics increased. In fact, the year 2015 marked a significant rise in the number of publications per year. Notably, two special issues devoted entirely to ELSI of epigenetics were published for the first time in 2015: one coordinated by philosopher of science Maurizio Meloni and published in the journal *New Genetics and Society*, the other coordinated by psychiatrists Tracy D Gunter and Alan R Felthous in *Behavioral Sciences & The Law*.

**Figure 1.**
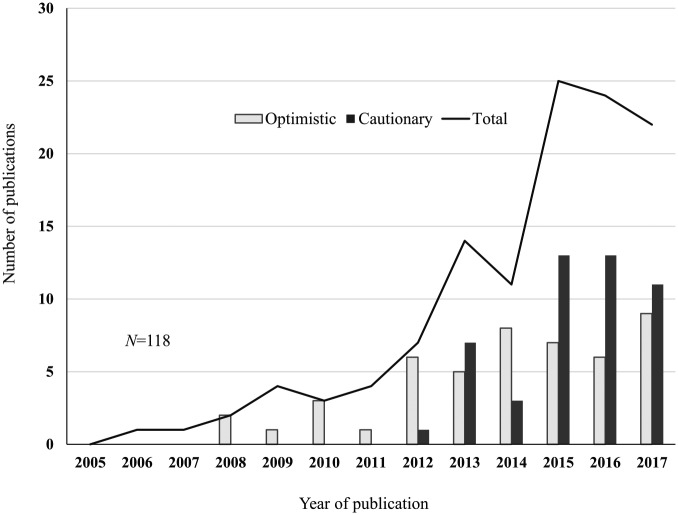
Number of publications per year. Total number of publications and according to the tone.

**Figure 2.**
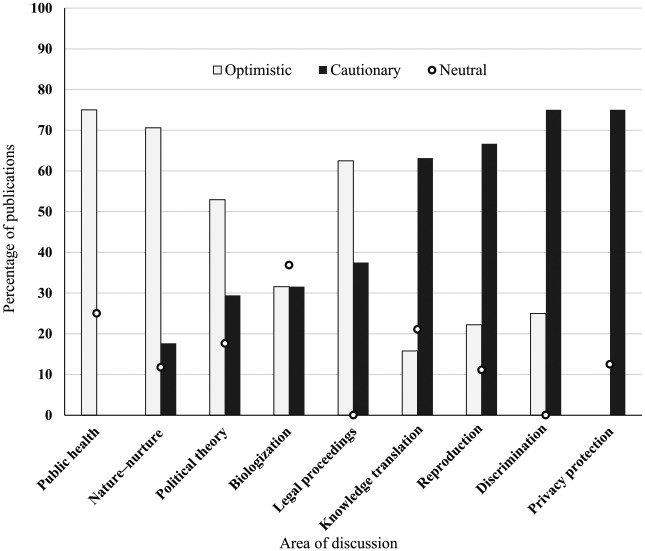
Proportion of optimistic, cautionary and neutral publications for each area of discussion.

While the first years were dominated by neutral and optimistic analyses of the ELSI of epigenetics, the past five years have witnessed a rise in cautionary analyses (Figure 1). The latter have emerged most notably regarding the protection of privacy and confidentiality in the collection, storage and sharing of epigenetic data, the risk of stigmatization and discrimination based on individual epigenetic information, and the potential impacts of epigenetics on reproduction and parenting (Figure 2). Cautionary claims were also prevalent in papers that investigated the translation of knowledge in epigenetics from the bench to the clinic and/or policy making field, and by authors who reflected on expectations and promissory discourses emerging with this new field of study. In other areas of discussion, there were more optimistic expectations toward epigenetics, including those related to its potential productive influence on the development and implementation of preventive public health strategies, and its anticipated positive impact on overdualistic views of the concepts of nature (biology) and nurture (family and culture). Taken together, these cautionary appraisals accounted for almost half of the papers published since 2013 (*n* = 47/96). While publications where authors enthusiastically promoted the potential applications of the field persisted over time, the overall proportion of these diminished over the past five years (*n* = 35/96).

We also found a relationship between areas of discussion and the analytical approach that the authors adopted (Table 1). For instance, the meaning of epigenetics for political theory (theories of justice and moral responsibility) appeared to stem from dialectical analyses of the properties of the biology under scrutiny (the impact of intrinsic contingencies). However, concerns about biologization, discrimination and knowledge translation arose following reflexive inquiries; that is, when exploring the possible effects of the broader context in which science is currently embedded (the impact of extrinsic contingencies). Such distinct analytical approaches may be related to the specific methods of studying the implications of science and technology inherent to the scholars’ academic disciplines, and to their respective main research interests.

**Table 1.** Number and proportion (%) of publications adopting each analytical approach (descriptive, instrumental, dialectical and reflexive), and according to each of the areas of discussion (AoD) identified.

AoD	Descriptive	Instrumental	Dialectical	Reflexive	Total	*Empirical*
Nature–nurture	3 (17.6)	4 (23.5)	4 (23.5)	6 (35.3)	17	
Biologization		5 (26.3)	4 (21.1)	**10 (52.6)**	19	*1*
Public Health	2 (16.3)	**10 (83.7)**			12	
Reproduction		4 (44.4)	2 (22.2)	3 (33.3)	9	*1*
Political theory	2 (11.8)	4 (23.5)	**10 (58.8)**	1 (5.9)	17	*1*
Legal proceedings	3 (37.7)	1 (12.5)	2 (25.0)	2 (25.0)	8	
Discrimination		2 (25.0)		**6 (75.0)**	8	
Privacy protection	**5 (62.5)**		3 (37.5)		8	*3*
Knowledge translation	2 (10.5)	1 (5.3)	1 (5.3)	**15 (78.9)**	19	*4*

Notes: When reporting the results of an empirical study, a publication was also noted as such. Highlighted in bold are numbers representing more than 50.0% of the total number of publications in the area.

All nine areas of discussion are summarized below. The sequential ordering does not reflect their prevalence in the literature. Rather, it is to optimize the narrative and facilitate transitions between sections by prioritizing theoretically foundational and explanatory issues.

### Nature–nurture: Epigenetics as a bridge between disciplines, or ‘boundary object’

Epigenetics has captured the attention of anthropologists, philosophers and sociologists, because it has potential to bridge the traditionally opposed concepts of ‘nature’ and ‘nurture’ (Guthman and Mansfield, 2012; Moore, 2017). By the same token, many authors perceive epigenetics as a unique opportunity to bring together researchers from long isolated disciplines into productive, joint investigations at the intersection of biology and culture (Meloni, 2014; Panofsky, 2015: 1106). According to these authors, epigenetics should be seen as a ‘boundary object’ and serve as an argument to promote interdisciplinary work and innovative forms of ‘co-laboration’. In other words, it should encourage researchers to acknowledge the strengths and advantages of other research disciplines, and to recognize limitations in the methods that they are most familiar with (Niewöhner, 2015).

On the one hand, epigenetics may serve as argument against gene-centric views of human identity and health, often referred to as ‘genetic essentialism’ and/or ‘genetic determinism’ (Burbano, 2006: 861; Canning, 2008; Gonon and Moisan, 2013: 29; Kuzawa and Sweet, 2009: 11; Lock, 2015: 152). On the other hand, epigenetics may also serve as argument against controversial claims that social and other environmental influences are more important for individuality (e.g. personality, gender, health) than is biology (Boniolo and Testa, 2012; Osborne, 2015). Certain scholars propose the use of ‘co-productionist’ frameworks to provide better accounts for the increasingly blurred line between ‘nature’ and ‘nurture’, and to bridge concepts and approaches that are seemingly irreconcilable at first sight, such as positivist (or essentialist) and social constructivist research methods (Klepfer, 2016; Maller, 2016; Meloni, 2016; Meloni and Testa, 2014: 445).

For some authors, epigenetics represents a fertile ground for revisiting the gene-centric ‘Modern Synthesis of Evolution’ (neo-Darwinism). According to these authors, evolutionary theories should integrate higher levels of adaptation, such as epigenetics, to theories of selection pressures. Rather than focusing solely on competition for long-term survival between ‘selfish genes’ (cf. Dawkins, 1976), an updated theory of evolution, consistent with epigenetics, should include ‘ecology-relevant’ accounts of heritability (Bossdorf et al., 2008). This would include the role of altruism and cooperation between individuals organized in society, such as the ‘prosocial view’ of evolution (Meloni, 2014: 594). In the ‘post-genomic era’ (Meloni, 2015: 129; Moore, 2017) the genome should be understood as ‘biosocial’ (Müller et al., 2017a), and the gene, which had long been defined as a chemically stable, fixed entity, should be reconceptualized as more ‘plastic’ and ‘reactive’ to its various environments than originally depicted.

Although epigenetics may shed light onto the reductionism of gene-centric theories, some authors are already concerned about the resurgence of Lamarckian accounts of heritability (Gadjev, 2015). Indeed, it is important to acknowledge that so far, there is only limited evidence of the transgenerational inheritance of a few epigenetic variants in humans. This limits interpretations about the persistence of acquired epigenetic traits across multiple generations. Additionally, there are emerging concerns about new forms of determinism that may arise with epigenetics (Landecker and Panofsky, 2013; Pickersgill et al., 2013; Tolwinski, 2013). According to other commentators, conceptualizing epigenetics as fundamentally ‘anti-determinist’ or ‘non-determinist’ could blind us to the potential rise of equally problematic forms of ‘environmental determinism’ (Landecker, 2010), or ‘epigenetic determinism’ (Mansfield and Guthman, 2015; Richardson, 2015; Waggoner and Uller, 2015: 178).

### Biologization: Epigenetics and the ‘molecularization of biography and milieu’

The epistemic upheaval proposed by epigenetics seems to reside in a certain valorization of the social, through a better understanding of its complex interrelations with, and influences on, biology (Jablonka, 2016; Lerner and Overton, 2017; Meloni and Testa, 2014: 449). Following developments in this field, environmental and sociocultural circumstances can be understood as external ‘signals’ (Landecker, 2016; Shields, 2017) which can be ‘mechanistically’ internalized into the body, thus actively producing long-term biochemical changes and becoming an integral part of a person’s ‘epigenetic history’ (Boniolo and Testa, 2012; Osborne, 2015). This ‘embodiment’ (Thayer and Non, 2015) of a person’s experiences and surroundings is conceptualized as the ‘molecularization of biography and milieu’ (Niewöhner, 2011: 291).

According to some authors, the new ‘biologization’ of social space and time may gain increasing traction. Eventually, it may become deeply integrated into individual and social discourses and practices under a novel ‘somatic sociality’ based on knowledge about epigenetic processes (Niewöhner, 2011: 292). On the one hand, research in the field of epigenetics could contribute to the reconfiguration of complex notions of social change and political movements into their corresponding standardized, material metrics at the biological level (Chung et al., 2016; Davis, 2014; Lloyd and Raikhel, 2018). Epigenetic markers could be mobilized by deprived individuals or groups as proof of past exposure to unfair shares of social adversity, and as an additional argument from the molecular level for greater social justice (Jaarsma, 2017; Kuzawa and Sweet, 2009; Sullivan, 2013: 190–218).

On the other hand, there could be adverse effects from translating social disparities into biological inequalities and reducing complex social problems to molecular codes (Blackman, 2016; Landecker and Panofsky, 2013; Lappé and Landecker, 2015: 153; Lickliter and Witherington, 2017). As observed by a few authors, simplistic deterministic thinking could be fueled, in the near future, by further evidence of (1) associations between some epigenetic variants and genotypes, (2) the persistence of epigenetic variants programmed during a child’s development, and (3) the possible transmission of some epigenetic variants across generations (Mansfield, 2017; Stelmach and Nerlich, 2015; Waggoner and Uller, 2015).

As acknowledged by many scholars, we should learn from the history of biologizing social phenomena (e.g. research in sociobiology) and remain cautious of its potential pitfalls (Müller et al., 2017b). To prevent the resurgence of ‘somatic reductionism’ (Lock, 2013) or ‘biological neo-reductionism’ (Lock, 2015: 151), and to guide further investigations in and of epigenetics, conceptual tools have been proposed, such as ‘customary biology’ (Niewöhner, 2011: 293–294), ‘localizing biology’ (Niewöhner, 2015: 234–235) and ‘bio-objectification’ (Svalastog and Damjanovicova, 2015). The notion of ‘*bio*habitus’, for example, is proposed as an alternative to Bourdieu’s habitus, as it better accounts for the close intertwining of social and biological capitals that recent research in social epigenetics highlight (Warin et al., 2015: 68–69). Such propositions may be related to the rise of a larger conceptual framework, consistent with epigenetics, termed ‘embodied constructivism’ (Meloni, 2015).

### Public health: Epigenetics as an argument for better preventive public policies

By allowing for a better mechanistic understanding of the developmental origins of health and disease (DOHaD), epigenetics has been optimistically mobilized by a number of authors to encourage policymakers towards using improved preventive strategies (Rial-Sebbag et al., 2016). Environmental epigenetics, for instance, appears to some commentators as instrumental in strengthening existing arguments in favor of improved environmental policies and regulations (Sagl et al., 2007; Turker, 2012).

By shedding light onto the close intertwining of the human body and its surroundings, epigenetics has been described as presenting an opportunity to question narrow, overly biomedical conceptions of the field of bioethics, to expand its scope to environmental and public health concerns (Dupras et al., 2014; Vilas Boas Reis and Torquato de Oliveira Naves, 2016). Borrowing from ecological genetics, new techniques for analyzing epigenetic variations in populations may help to gather the evidence needed to justify these changes (Bäumen and Brand, 2010; Bossdorf et al., 2008).

Social epigenetics is also expected to provide new convincing arguments for promoting social policies. For instance, it may demonstrate how early life experiences can influence gene expression later in life, allowing for the development of policies that could improve children’s future health (McBride and Koehly, 2017; Park and Kobor, 2015). Moreover, epigenetics may help to demonstrate the value of social workers in public health, increasing the appreciation and funding of the profession (Combs-Orme, 2013). According to authors with these expectations, placing more attention on the social determinants of health may help to prevent a wide variety of health-related conditions, including anxiety and mental health (Lang et al., 2016).

According to many authors, the potential heritability of some epigenetic variants is increasing the stakes of these questions (Drake and Liu, 2010; Kabasenche and Skinner, 2014; Vilas Boas Reis and Torquato de Oliveira Naves, 2016). If epigenetic harm from toxic exposures or social adversity is likely to also affect future generations, then using preventive strategies may be more of an urgent priority than before. The potential epigenetic inheritance of obesity-related diseases, for instance, is now understood as a significant concern for public health in the light of recent advances in epigenetics (Niculescu, 2011). According to other scholars, however, the significance of epigenetics to public health should not be overstated, considering the still nascent state of the field. Additionally, they argue that there is a current absence of compelling evidence of epigenetic inheritance in humans and a high probability of confounding variables in environmental and social epigenetics studies, and that there are important meta-ethics questions relating to the degree of normative/prescriptive value that should be granted to empirical findings (Chung et al., 2016; Huang and King, 2017; Juengst et al., 2014).

### Reproduction: The mother as ‘epigenetic vector’ of children’s health

Scientific studies on the potential transmission of epigenetic risks from parent to child, effects of the in utero environment on epigenetic programming, as well as the impact of the familial situation and parental behavior on the child’s future mental health, have brought the field to the forefront of sensitive conversations about human reproduction (Crawford, 2016). Cautionary analyses have arisen regarding the focus of many epigenetic studies on parental duties toward the future epigenetic health of their child at an individual level. Most notably, authors criticize epigenetics for its potential to place additional blame on mothers who adopt specific behaviors during pregnancy or as caregivers (Hens, 2017; Kenney and Muller, 2017; Richardson, 2015), encouraging a new sense of maternal ‘hyper-responsibility’ (Lafaye, 2014; M’hamdi et al., 2018; Warin et al., 2015).

Many suggest, for instance, that assisted reproductive technologies (ARTs) may influence epigenetic programming in a way that affects the future health of the children conceived. These studies raise important questions related to the ethical tension between harm reduction in children and reproductive autonomy, as ARTs can sometimes represent the only avenue for couples trying to conceive (Roy et al., 2017). Surrogacy has also come under scrutiny, as epigenetic research now suggests that the genetic makeup and environment of a surrogate can substantially impact gene expression in the child (Nicholson and Nicholson, 2016). Consequently, some authors are worried that the ‘foregrounding of the maternal body as an epigenetic vector’ could lead to increased manipulation and control over the female body (Richardson, 2015).

A paucity of evidence on the implications of epigenetic modifications at different points in the life cycle has compelled researchers to exercise caution when making claims about emerging findings on DOHaD (Geronimus, 2013; Huang and King, 2017). Such concerns are exacerbated by the challenges of translating findings from animal studies into the implications for human reproduction. Specifically, some authors expressed concerns that these studies are being inaccurately extrapolated to human behavior in ways that reinforce existing sex stereotypes (Kenney and Muller, 2017; Richardson, 2015). In fact, rat studies that examine maternal behavior and its impact on their offspring are already being used to imply similar consequences in human mother–child interactions. Moreover, by centering the discussions of epigenetic programming on the mother’s body and behaviors, much of this research largely ignores the role of fathers in terms of both inherited and child-rearing epigenetic contributions (Hens, 2017; Richardson, 2015).

Other scholars have nevertheless expressed optimism about new understandings of the breadth of what can be considered ‘maternal exposure’. Taking into account mothers’ lives *prior* to child-bearing, rather than focusing only on women’s individual actions during their reproductive windows, may help to better support the health of vulnerable populations throughout the lifespan (M’hamdi et al., 2017; Shields, 2017). By providing evidence that public policies aimed at improving social and economic factors can have tangible impacts on biology, epigenetic research can affect the direction of health interventions while simultaneously respecting the best interests of parents and children (Wallack and Thornburg, 2016; White and Wastell, 2017).

### Political theory: Epigenetic perspectives on theories of justice and moral responsibility

As mentioned, epigenetics has been used to show that the disproportionate exposures of some groups to pollutants and/or social adversity can help explain biological inequalities between persons, and possibly even between generations. Moreover, it has been used as a tool to help claim that such unfair health disparities could, and arguably should, be prevented through social policy. These discussions have most often been framed, respectively, with the principles of ‘environmental justice’ and ‘intergenerational equity’ (Butkus and Kolmes, 2017; Rothstein et al., 2009a, 2009b: 25, 2017).

Authors have consistently discussed the meaning of epigenetics for theories of justice. For instance, some authors have questioned the traditional opposition between Rawlsian approaches, focused solely on socially induced disparities in life opportunities, and luck-egalitarian theories of justice, which also consider innate/inherited biological inequalities that unfairly reduce life opportunities immediately at birth. By shedding light on the ways that social injustices are being embodied and even transferred to children (Erwin, 2015: 670), epigenetics would be blurring the line between the so-called ‘social lottery’ (i.e. socially induced endowments) and the ‘genetic lottery’ (i.e. biological endowments). It would also, at the same time, blur the line between the two abovementioned theories of justice (Lafaye, 2014; Loi et al., 2013; Meloni, 2015).

Most authors predominantly stressed an important role states have in preventing epigenetic risks (Combs-Orme, 2013; Dupras et al., 2014; Gesche, 2010: 285; Hedlund, 2012; Lafaye, 2014: 5; Shrader-Frechette, 2012). However, there are an increasing number of dialectical analyses on epigenetic responsibilities in the scholarly literature, which point to a possible tension between collective and individual moral responsibilities for epigenetic health (Chiapperino and Testa, 2016; Dupras and Ravitsky, 2016a; Evers, 2014; Evers and Changeux, 2016; Meloni and Testa, 2014: 444; Niewöhner, 2015: 224). For instance, there are emerging discussions about to what extent, and under which circumstances, individuals have the capacity to modulate their own and their child’s epigenetic risks (Boniolo, 2013: 408). However, many authors note the difficulty in determining with certainty *which* epigenetic harms have been acquired or imposed on others following voluntary actions (Erwin, 2015; Gunter and Felthous, 2015; Niculescu, 2011: G23; Tamatea, 2015: 639).

Challenges also arise when attempting to identify ‘reference’ epigenomes and with subsequently defining the ‘healthy’ epigenomes. In fact, it is not always easy to distinguish between epigenetic variants that should be considered biological disruptions leading to higher risks for some diseases, and those that are advantageous biological adaptations to specific developmental contexts (Stapleton et al., 2012: 135). Moreover, the variable stability and dynamism/reversibility of diverse types of epigenetic modifications, in different cells and at different periods of time, introduce additional difficulties in the attribution of moral epigenetics responsibilities (Chadwick and O’Connor, 2013: 464; Dupras and Ravitsky, 2016a; Vears and D’Abramo, 2018). The ‘non-identity problem’ – a philosophical conundrum stemming from the meta-ethical question of whether it is possible to harm someone who does not yet exist (i.e. preconception) – is also discussed as a foreseeable limit to our duties toward the epigenetic health of future generations (Del Savio et al., 2015; Kabasenche and Skinner, 2014; Roy et al., 2017).

### Legal proceedings: Epigenetic markers as evidence of causality in tort law

According to some legal scholars, epigenetics research may affect legal proceedings in the future, by providing additional evidentiary input into the realm of civil liability (Khan, 2010; Turker, 2012; Vandenbergh et al., 2017). ‘Tort law’ refers to situations where one person’s ‘wrong’ causes another person’s loss or harm, resulting in legal liability for the tortious act. This liability is calculated using three components: act, injury and causation. Causation has been the most difficult aspect to prove in the courtroom, where judges have generally been reluctant to rely on population studies – which show statistically significant associations between specific acts and observed injuries – as convincing evidence of causation.

According to some authors, epigenetics may help to bridge this evidence gap by providing information about the molecular mechanisms that link, for instance, exposure to chemicals (e.g. pollutants, cosmetics, drugs) and the occurrence of diseases. By so doing, epigenetic studies would be able to offer claimants valuable complementary evidence of civil liability potentially admissible in court (Laubach, 2016). The ability to trace epigenetic harms directly back to their cause presents new opportunities not only to provide compensation to victims of environmental harms, but also in developing new regulatory schemes and policies that better reflect our new understanding of the effects of toxins on the body (Turker, 2012; Vandenbergh et al., 2017). Moreover, authors discuss the possibility that more precise evidence of epigenetic harm due to negligent parenting could give rise to new legal repercussions for preconception and periconception parental behaviors (Wiener, 2010).

Despite overall optimism about the potential use of epigenetic evidence in toxic torts, legal scholars recognize a number of barriers to its implementation (Khan, 2010). First, the long latency period between the harmful act and the emergence of symptoms from the harm may fall afoul of the statutes of limitations. Additionally, lack of access to both the judicial system and the necessary epigenetic evidence may impede vulnerable parties from making full use of these innovations in tort law. Finally, the difficulty of quantifying epigenetic harm with certainty will complicate the applications in the courtroom. All these risks, however, may be mitigated by updating laws, regulations and policies to recognize these new kinds of evidence, if necessary (Khan, 2010; Rothstein, 2013).

So far, the potential implications of epigenetics for criminal law have not yet received the same attention as for tort law, though this nevertheless is an area where we can expect to see further implications unfold as the field progresses. In advancing our understanding of the long-term neurological and psychological impacts of trauma, for instance, epigenetic research may alter our understanding of criminal responsibility (Gunter and Felthous, 2015). In reconceptualizing conditions that have been traditionally considered behavioral disorders (e.g. psychopathy, sociopathy) as biological disorders caused by toxic exposures or adverse experiences, or previous epigenetic harm, epigenetics could impact both criminal risk prediction and the ways in which we punish, prevent and/or treat such behaviors (Tamatea, 2015).

### Discrimination: Epigenetics as opportunity or risk for underserved populations?

Some scholars have observed that epigenetic research may help us better understand the biological pathways through which some of the negative effects of unfair social structures and discrimination can persist over generations (Sullivan, 2013). For instance, researchers suggest an epigenetic model of racial health disparities with regard to the marked prevalence of premature birth and cardiovascular illness among African-Americans (Kuzawa and Sweet, 2009). This model has been promoted as an opportunity for marginalized and underserved populations to claim compensation for the harmful and long-lasting consequences of disproportionate levels of toxic environmental exposures and social adversity with which these communities battle.

New epigenetic understandings of social disparities, however, have led a few authors to raise ethical issues inherent to making biological comparisons between social groups. For instance, authors discuss the risks associated with the reification of the idea that biological races exist (Pickersgill et al., 2013; Saulnier and Dupras, 2017). Epigenetic research may offer new insights into the long-term consequences of discriminatory attitudes, discourses, practices and/or social structures on the biology and health of discriminated individuals and populations. However, some fear that the emergence of reductionist and fatalist views of embodiment could lead to increased ‘racialization’ and to the interpretation that some vulnerable groups may be too epigenetically damaged to be rescued through preventive public policies (Katz, 2013; Meloni, 2017). Heeding lessons from genomics and the history of biological research creating or reinforcing stereotypes (Graham, 2016), many authors involved in this area have subsequently adopted a cautionary tone when measuring and discussing social disparities using biological metrics (Guthman and Mansfield, 2012; Kuzawa and Sweet, 2009).

More specifically, research in epigenetics raises concerns regarding the normalization of privileged bodies (e.g. measuring obesity based on norms seen in white bodies). The potential for environmental epigenetics to allow the measurement of biomarkers of social inequality in new ways underscores the concerns raised across categories about the development of new measurements of ‘normality’. This could lead to preventive policies that target the health of vulnerable populations in a form of ‘epi-eugenics’ (Juengst et al., 2014). Accordingly, racialized populations, women, sexual minorities and socioeconomically underprivileged individuals face the risk of more intense scrutiny and increased blame for sociocultural practices or behaviors that deviate from the new epigenetic norm (Katz, 2013; Lock, 2013; Niewöhner, 2015).

Epigenetic evidence of the DOHaD is seen as promising for preventive public policies. However, many fear that some of these policies may disproportionally burden already vulnerable groups, by normalizing epigenetically favorable environments and further marginalizing others (Mansfield, 2012; Mansfield and Guthman, 2015). Authors suggest there ought to be a balance between recognizing the problems generated by past racialized research, and the potential for a new ‘plastic and biosocial’ view of race that draws together social and biological evidence of harm (Jaarsma, 2017; Meloni, 2017).

### Privacy protection: Adapting the degree of protection according to the risk level

Some authors are concerned about the protection of privacy and confidentiality of research participants involved in epigenetic research. Most of them associated their concerns about privacy with the previously discussed issue of discrimination, reflecting on their worry that epigenetic information, if not treated with the necessary caution, could result in further stigmatization and/or adverse differential treatment of groups (Dyke et al., 2015; Erwin, 2015; Terry, 2015; Thomas, 2015). In a way, individual epigenetic data could prove to be even more ethically sensitive than genetic data, considering that it can provide information not only about an individual’s disease risk profile – and sometimes on the current disease status – but also on the individual’s previous exposures and lifestyle (Backes et al., 2016).

Moreover, uncertainties persist about the applicability of epigenetics to existing legal and normative frameworks that were designed to protect the privacy of individual genetic information. It is unclear whether the differences in biological properties between epigenetics and genetics necessitates new ethical guidelines and legal frameworks, or whether already existing policies could be sufficient or be adapted for the protection of individual epigenetic data (Rothstein, 2013; Thomas, 2015). Although most publications in this category were found to be cautionary in tone, there are disagreements regarding the level of protection of epigenetic data required to protect the privacy of patients and/or research participants.

Misconceptions about the identifiability of certain types of epigenetic information may lead to an under- or overestimation of the associated privacy risks. For instance, although it is commonly believed that the ‘inherent temporal variability of microRNAs (miRNAs)’ protects the data from being tracked and linked to an individual, a specific study found that miRNA expression profiles in blood could in fact be matched to a specific individual with a success rate of 90% (Backes et al., 2016). Reflecting on this same concern, another study showed that certain sensitive information about research participants could be linked to individuals using epigenetic datasets that were being shared online (Philibert et al., 2014).

Other authors, however, are concerned that overly restricting access to epigenetic data, without appropriate assessments of the actual probabilities of privacy breach (which may vary according to the specific dataset and context of the study), could unreasonably hinder the progress of epigenetics research (Dyke et al., 2015; Joly et al., 2015). According to them, the level of protection of privacy should be commensurate with a fair evaluation of the risks. Epigenetic studies on vulnerable populations, for instance, are generally thought to necessitate special cautions, especially when the populations are associated with specific geographical locations or ethnicities.

### Knowledge translation: Critical analyses of emerging discourses

In the past five years, there has been an increase in the number of publications by scholars reflecting on the contingencies, significations and possible limitations of previous and ongoing appraisals of the potential ELSI of epigenetics. These authors – mostly social scientists – provide important critical analyses on promissory or alarmist discourses that are currently emerging within this field, and on the metaphors that are being used to illustrate its novelty (e.g. its differences compared with genetics) (Stelmach and Nerlich, 2015). The ‘revolutionary’ aspects of epigenetics, and the mobilization of its rhetorical power for ideological, political or commercial purposes, have been particularly scrutinized (Deichmann, 2016; Gadjev, 2015; Landecker and Panofsky, 2013; Mansfield, 2012; Meloni and Testa, 2014; Müller et al., 2017b; Pentecost and Cousins, 2017; Robison, 2016).

Some empirical studies have examined the views of researchers working in epigenetics. Semi-structured interviews reveal high variability in nature and degree of expectations toward the field, in light of persisting scientific uncertainties (Tolwinski, 2013). Another similar study cautioned the possible role of social scientists working alongside overenthusiastic lab scientists in overstating the accumulated scientific evidence. When anticipating the significance of epigenetics for society, they would contribute to disrupting the message that is sent to the public about the real versus hypothetical ELSI of epigenetics (Joly et al., 2016; Pickersgill, 2016). Studies have also identified the media as having an important responsibility in emerging problems related to knowledge transfer (Gonon and Moisan, 2013; Juengst et al., 2014; Lappé, 2016). It thus appears important that scientists, health care providers, governments and citizens engage with the new epigenetic knowledge (Gesche, 2010), and get involved in setting standards for adequate communication of epigenetic information, for instance with patients and/or research participants (Belrhomari, 2014; Rial-Sebbag et al., 2016; Roy et al., 2017).

Some authors also highlight the need to further investigate and address subtle biases and barriers that may impede proper translation of scientific findings into fair and effective health interventions (Pickersgill et al., 2013; 2014). As such, the subjective perspectives and different interests of stakeholders should be acknowledged (Sullivan, 2013). Among other types of possible influences, financial pressures by private actors to commodify research findings and to commercialize clinical applications of epigenetics could impede other types of translations of epigenetics. For instance, they could impede collective preventive strategies aimed at improving public health more directly by tackling environmental injustices and social inequalities (Dupras and Ravitsky, 2016b; Niewöhner, 2011).

## Discussion

In this article, we have provided a multidisciplinary landscape of the current discussions about the ELSI of epigenetics. Aware of critics who have shed light on the problems of narrow ELSI approaches, and inspired by more inclusive frameworks in ‘responsible research and innovation’ (Balmer et al., 2015, 2016; Myskja et al., 2014; Wickson and Carew, 2014), we chose to not only place attention on consequentialist analyses traditionally included under the umbrella of ELSI programs (e.g. framed by the four moral principles of respect for autonomy, nonmalfeasance, beneficence and justice), but also to expand our analysis to include hermeneutic analyses of foundational epistemic questions raised by epigenetics in the post-genomic era (see Grunwald, 2017). We thus included, for instance, discussions about the expected impact of epigenetics on the nature versus nurture dualism, for their potential to influence interpretations of the meaning of epigenetics for health and society.

In the same vein, we found it important to report all the potential *implications* of the field, rather than focusing solely on expected *issues*. This was to account not only for the potential undesirable outcomes anticipated by some scholars, but also for enthusiastic expectations towards epigenetics. This inclusion allowed us to characterize this emerging field as a site of contestation, rich with a variety of disciplinary perspectives and analytical approaches. We thus characterized nine recurrent ‘areas of discussion’ and pointed to analyses in tension with one another, by different authors arguing for different perspectives or positions between and within each of these areas. In doing so, we revealed high heterogeneity in focus and interpretation in this emerging field.

As we noted, early appraisals of the ELSI of epigenetics often led to analyses about the opportunities this field may offer when contrasted with genetics, rather than in-depth analyses of risks and tensions arising within the field. A rather simple yet possible explanation for this could be that, at that time, discussions were still in their infancy, and that it was too early for scholars to reflect on the relevance, adequacy and pitfalls of previous interpretations of the implications of epigenetics put forth by their peers. Another explanation, which we tend to lean toward, is that the genetic era, and its exaggerated emphasis on the biological sources of identity, behavior and health, had created some sort of vacuum for any molecular-scale evidence that would reinvigorate the epistemic status of social sciences and humanities. Paradoxically, social and political theories were *in need* of biological support (see Dupras, 2017), in order to be able to counterstrike ongoing geneticization and biomedicalization processes on the same epistemic battlefield (see Dupras et al., 2016b); this is what epigenetics was finally offering.

At first glance, epigenetics can appear as the perfect ‘anti-genetics’ instrument. As many authors later noted, however, it may not be all so simple. Since 2013, we have witnessed an increasingly sophisticated body of literature, with a growing number of publications beginning to adopt a cautionary tone regarding scientific developments in epigenetics and emerging claims about the field’s epistemological and normative significance. The growing diversity in tone and analytical approach was observed not only across publications and according to the main area of discussion of a paper, but also, within publications themselves, sometimes with the same author(s) stating both potential opportunities and risks associated with scientific developments in epigenetics.

We would like to argue that the coexistence of descriptive, instrumental, dialectical and reflexive analyses of the potential implications of a new scientific field or biotechnology is a sign of sound interdisciplinary conversations. Instrumental analyses of epigenetics have been portrayed as exaggerated and overly enthusiastic. While this may sometimes be the case, we believe that instrumental analyses are sometimes necessary to fill pre-existing gaps in scientific knowledge, and to rectify related epistemic injustices that such gaps had created, affecting different groups of stakeholders in the process (Dupras et al., 2017). Sometimes a vacuum has built, and it must be filled. This is possibly what scholars claiming a ‘post-genomic’ era *for* the social sciences and humanities have been attempting to achieve.

Over the past few years, new discussions have emerged, for example regarding when and how scientifically valid and clinically actionable epigenetic information should be communicated to patients or returned to research participants (Dyke et al., 2019; Rial-Sebbag et al., 2016; Roy et al., 2017). In this article, these recent concerns were categorized under the area of discussion ‘knowledge translation’. However, discussions in the future about such issues could be categorized under another area specific to ‘communication and counseling’. Indeed, a set of implications that was found to be largely absent from the reviewed literature relates to questions about consent to epigenetic interventions or research. This is surprising, considering the prominent place that has been granted to the principle of respect for autonomy in genetics and in the bioethics literature more generally over the past 50 years.

Such discussions could become more prevalent in the coming years, as the clinical and public health utility of interventions building on epigenetic data are scientifically validated, and as the field’s implications become recognized as diverging in some regards and to some extent from those of genetics. It will be interesting, for instance, to investigate further whether the content of consent forms in epigenetics research should be adapted to account for the specificities of epigenetic mechanisms and information. Recently, private companies such as Yousurance^TM^ (for life insurance underwritings) and Chronomics^TM^ (for recreational purposes) have started to advertise and offer epigenetic tests directly to consumers online. It remains to be seen whether these companies are offering adequate support to their consumers to promote free and informed consent when they decide to undergo these tests. Potential (mis)use of the results of epigenetics tests by both private companies (e.g. insurance) and public agencies (e.g. forensics, immigration) will also deserve close ethical and legal scrutiny in the coming years (Dupras et al., 2018).

It should appear evident following this review that it is the multidisciplinary nature of the emerging ELSI literature that is at the source of a rich and nuanced body of discussion around the significance of epigenetics for health and society. These discussions can be sometimes portrayed as conflictual oppositions between authors from different epistemological standpoints (Panofsky, 2015), and with different sorts of relationships with normativity (see Chiapperino, 2018; Huang and King, 2017). Nonetheless, we argue that it is the cumulated effect of descriptive, instrumental, dialectical and reflexive analyses of the potential implications of science and technology which ultimately produces sophistication in this new and exciting field of study at the crossroads of epigenetics, ethics, law and society.

Of course, it is crucial to keep in mind that the current epigenetics ELSI literature is mostly anticipatory and speculative (Juengst et al., 2014). With further developments in epigenetics, it will be important to validate or invalidate the different hypothetical scenarios with more empirical evidence from both biological and social sciences. Interdisciplinary studies that recognize the coexistence of distinct analytical approaches and celebrate their complementary contributions to the richness of social sciences and humanities will be key to assessing and addressing the ELSI of epigenetics in the coming years.

## Supplemental Material

Appendix – Supplemental material for Epigenetics, ethics, law and society: A multidisciplinary review of descriptive, instrumental, dialectical and reflexive analyses Click here for additional data file. Supplemental material, Appendix for Epigenetics, ethics, law and society: A multidisciplinary review of descriptive, instrumental, dialectical and reflexive analyses by Charles Dupras, Katie Michelle Saulnier and Yann Joly in Social Studies of Science
