# Supplementary material for: Epigenetics, ethics, law and society: A multidisciplinary review of descriptive, instrumental, dialectical and reflexive analyses
Source: Soc Stud Sci. 2019 Aug 1;49(5):785–810. doi: 10.1177/0306312719866007 (PMC6801799; doi:10.1177/0306312719866007)
Supplement: Appendix – Supplemental material for Epigenetics, ethics, law and society: A multidisciplinary review of descriptive, instrumental, dialectical and reflexive analyses [file Appendix.pptx]

## Slide 1
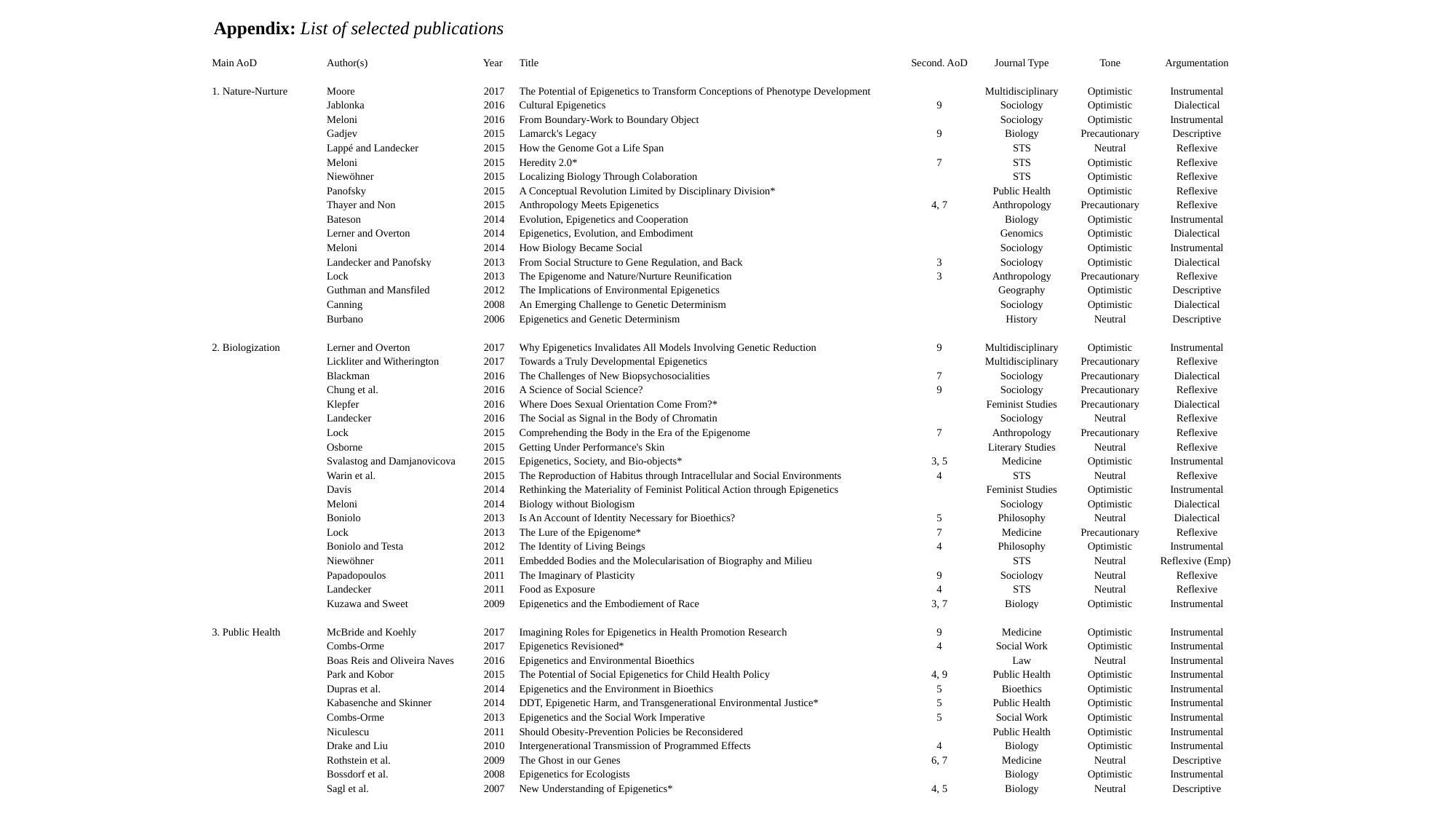

Appendix: List of selected publications
| Main AoD | Author(s) | Year | Title | Second. AoD | Journal Type | Tone | Argumentation |
| --- | --- | --- | --- | --- | --- | --- | --- |
| | | | | | | | |
| 1. Nature-Nurture | Moore | 2017 | The Potential of Epigenetics to Transform Conceptions of Phenotype Development | ­ | Multidisciplinary | Optimistic | Instrumental |
| | Jablonka | 2016 | Cultural Epigenetics | 9 | Sociology | Optimistic | Dialectical |
| | Meloni | 2016 | From Boundary-Work to Boundary Object | ­ | Sociology | Optimistic | Instrumental |
| | Gadjev | 2015 | Lamarck's Legacy | 9 | Biology | Precautionary | Descriptive |
| | Lappé and Landecker | 2015 | How the Genome Got a Life Span | ­ | STS | Neutral | Reflexive |
| | Meloni | 2015 | Heredity 2.0\* | 7 | STS | Optimistic | Reflexive |
| | Niewöhner | 2015 | Localizing Biology Through Colaboration | ­ | STS | Optimistic | Reflexive |
| | Panofsky | 2015 | A Conceptual Revolution Limited by Disciplinary Division\* | ­ | Public Health | Optimistic | Reflexive |
| | Thayer and Non | 2015 | Anthropology Meets Epigenetics | 4, 7 | Anthropology | Precautionary | Reflexive |
| | Bateson | 2014 | Evolution, Epigenetics and Cooperation | ­ | Biology | Optimistic | Instrumental |
| | Lerner and Overton | 2014 | Epigenetics, Evolution, and Embodiment | ­ | Genomics | Optimistic | Dialectical |
| | Meloni | 2014 | How Biology Became Social | ­ | Sociology | Optimistic | Instrumental |
| | Landecker and Panofsky | 2013 | From Social Structure to Gene Regulation, and Back | 3 | Sociology | Optimistic | Dialectical |
| | Lock | 2013 | The Epigenome and Nature/Nurture Reunification | 3 | Anthropology | Precautionary | Reflexive |
| | Guthman and Mansfiled | 2012 | The Implications of Environmental Epigenetics | ­ | Geography | Optimistic | Descriptive |
| | Canning | 2008 | An Emerging Challenge to Genetic Determinism | ­ | Sociology | Optimistic | Dialectical |
| | Burbano | 2006 | Epigenetics and Genetic Determinism | ­ | History | Neutral | Descriptive |
| | | | | | | | |
| 2. Biologization | Lerner and Overton | 2017 | Why Epigenetics Invalidates All Models Involving Genetic Reduction | 9 | Multidisciplinary | Optimistic | Instrumental |
| | Lickliter and Witherington | 2017 | Towards a Truly Developmental Epigenetics | ­ | Multidisciplinary | Precautionary | Reflexive |
| | Blackman | 2016 | The Challenges of New Biopsychosocialities | 7 | Sociology | Precautionary | Dialectical |
| | Chung et al. | 2016 | A Science of Social Science? | 9 | Sociology | Precautionary | Reflexive |
| | Klepfer | 2016 | Where Does Sexual Orientation Come From?\* | ­ | Feminist Studies | Precautionary | Dialectical |
| | Landecker | 2016 | The Social as Signal in the Body of Chromatin | ­ | Sociology | Neutral | Reflexive |
| | Lock | 2015 | Comprehending the Body in the Era of the Epigenome | 7 | Anthropology | Precautionary | Reflexive |
| | Osborne | 2015 | Getting Under Performance's Skin | ­ | Literary Studies | Neutral | Reflexive |
| | Svalastog and Damjanovicova | 2015 | Epigenetics, Society, and Bio-objects\* | 3, 5 | Medicine | Optimistic | Instrumental |
| | Warin et al. | 2015 | The Reproduction of Habitus through Intracellular and Social Environments | 4 | STS | Neutral | Reflexive |
| | Davis | 2014 | Rethinking the Materiality of Feminist Political Action through Epigenetics | ­ | Feminist Studies | Optimistic | Instrumental |
| | Meloni | 2014 | Biology without Biologism | ­ | Sociology | Optimistic | Dialectical |
| | Boniolo | 2013 | Is An Account of Identity Necessary for Bioethics? | 5 | Philosophy | Neutral | Dialectical |
| | Lock | 2013 | The Lure of the Epigenome\* | 7 | Medicine | Precautionary | Reflexive |
| | Boniolo and Testa | 2012 | The Identity of Living Beings | 4 | Philosophy | Optimistic | Instrumental |
| | Niewöhner | 2011 | Embedded Bodies and the Molecularisation of Biography and Milieu | ­ | STS | Neutral | Reflexive (Emp) |
| | Papadopoulos | 2011 | The Imaginary of Plasticity | 9 | Sociology | Neutral | Reflexive |
| | Landecker | 2011 | Food as Exposure | 4 | STS | Neutral | Reflexive |
| | Kuzawa and Sweet | 2009 | Epigenetics and the Embodiement of Race | 3, 7 | Biology | Optimistic | Instrumental |
| | | | | | | | |
| 3. Public Health | McBride and Koehly | 2017 | Imagining Roles for Epigenetics in Health Promotion Research | 9 | Medicine | Optimistic | Instrumental |
| | Combs-Orme | 2017 | Epigenetics Revisioned\* | 4 | Social Work | Optimistic | Instrumental |
| | Boas Reis and Oliveira Naves | 2016 | Epigenetics and Environmental Bioethics | ­ | Law | Neutral | Instrumental |
| | Park and Kobor | 2015 | The Potential of Social Epigenetics for Child Health Policy | 4, 9 | Public Health | Optimistic | Instrumental |
| | Dupras et al. | 2014 | Epigenetics and the Environment in Bioethics | 5 | Bioethics | Optimistic | Instrumental |
| | Kabasenche and Skinner | 2014 | DDT, Epigenetic Harm, and Transgenerational Environmental Justice\* | 5 | Public Health | Optimistic | Instrumental |
| | Combs-Orme | 2013 | Epigenetics and the Social Work Imperative | 5 | Social Work | Optimistic | Instrumental |
| | Niculescu | 2011 | Should Obesity-Prevention Policies be Reconsidered | ­ | Public Health | Optimistic | Instrumental |
| | Drake and Liu | 2010 | Intergenerational Transmission of Programmed Effects | 4 | Biology | Optimistic | Instrumental |
| | Rothstein et al. | 2009 | The Ghost in our Genes | 6, 7 | Medicine | Neutral | Descriptive |
| | Bossdorf et al. | 2008 | Epigenetics for Ecologists | ­ | Biology | Optimistic | Instrumental |
| | Sagl et al. | 2007 | New Understanding of Epigenetics\* | 4, 5 | Biology | Neutral | Descriptive |

## Slide 2
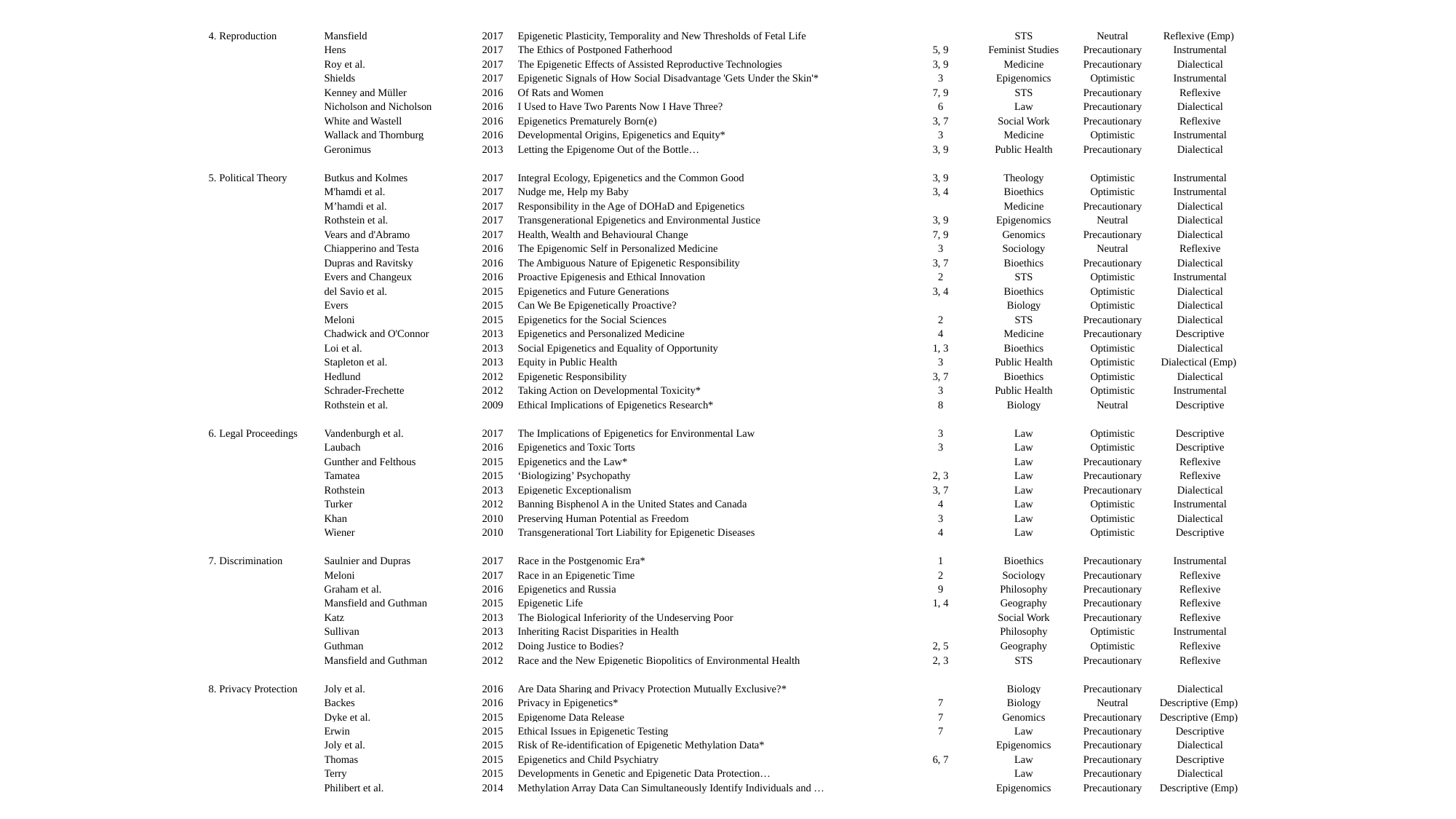

| 4. Reproduction | Mansfield | 2017 | Epigenetic Plasticity, Temporality and New Thresholds of Fetal Life | ­ | STS | Neutral | Reflexive (Emp) |
| --- | --- | --- | --- | --- | --- | --- | --- |
| | Hens | 2017 | The Ethics of Postponed Fatherhood | 5, 9 | Feminist Studies | Precautionary | Instrumental |
| | Roy et al. | 2017 | The Epigenetic Effects of Assisted Reproductive Technologies | 3, 9 | Medicine | Precautionary | Dialectical |
| | Shields | 2017 | Epigenetic Signals of How Social Disadvantage 'Gets Under the Skin'\* | 3 | Epigenomics | Optimistic | Instrumental |
| | Kenney and Müller | 2016 | Of Rats and Women | 7, 9 | STS | Precautionary | Reflexive |
| | Nicholson and Nicholson | 2016 | I Used to Have Two Parents Now I Have Three? | 6 | Law | Precautionary | Dialectical |
| | White and Wastell | 2016 | Epigenetics Prematurely Born(e) | 3, 7 | Social Work | Precautionary | Reflexive |
| | Wallack and Thornburg | 2016 | Developmental Origins, Epigenetics and Equity\* | 3 | Medicine | Optimistic | Instrumental |
| | Geronimus | 2013 | Letting the Epigenome Out of the Bottle… | 3, 9 | Public Health | Precautionary | Dialectical |
| | | | | | | | |
| 5. Political Theory | Butkus and Kolmes | 2017 | Integral Ecology, Epigenetics and the Common Good | 3, 9 | Theology | Optimistic | Instrumental |
| | M'hamdi et al. | 2017 | Nudge me, Help my Baby | 3, 4 | Bioethics | Optimistic | Instrumental |
| | M’hamdi et al. | 2017 | Responsibility in the Age of DOHaD and Epigenetics | ­ | Medicine | Precautionary | Dialectical |
| | Rothstein et al. | 2017 | Transgenerational Epigenetics and Environmental Justice | 3, 9 | Epigenomics | Neutral | Dialectical |
| | Vears and d'Abramo | 2017 | Health, Wealth and Behavioural Change | 7, 9 | Genomics | Precautionary | Dialectical |
| | Chiapperino and Testa | 2016 | The Epigenomic Self in Personalized Medicine | 3 | Sociology | Neutral | Reflexive |
| | Dupras and Ravitsky | 2016 | The Ambiguous Nature of Epigenetic Responsibility | 3, 7 | Bioethics | Precautionary | Dialectical |
| | Evers and Changeux | 2016 | Proactive Epigenesis and Ethical Innovation | 2 | STS | Optimistic | Instrumental |
| | del Savio et al. | 2015 | Epigenetics and Future Generations | 3, 4 | Bioethics | Optimistic | Dialectical |
| | Evers | 2015 | Can We Be Epigenetically Proactive? | ­ | Biology | Optimistic | Dialectical |
| | Meloni | 2015 | Epigenetics for the Social Sciences | 2 | STS | Precautionary | Dialectical |
| | Chadwick and O'Connor | 2013 | Epigenetics and Personalized Medicine | 4 | Medicine | Precautionary | Descriptive |
| | Loi et al. | 2013 | Social Epigenetics and Equality of Opportunity | 1, 3 | Bioethics | Optimistic | Dialectical |
| | Stapleton et al. | 2013 | Equity in Public Health | 3 | Public Health | Optimistic | Dialectical (Emp) |
| | Hedlund | 2012 | Epigenetic Responsibility | 3, 7 | Bioethics | Optimistic | Dialectical |
| | Schrader-Frechette | 2012 | Taking Action on Developmental Toxicity\* | 3 | Public Health | Optimistic | Instrumental |
| | Rothstein et al. | 2009 | Ethical Implications of Epigenetics Research\* | 8 | Biology | Neutral | Descriptive |
| | | | | | | | |
| 6. Legal Proceedings | Vandenburgh et al. | 2017 | The Implications of Epigenetics for Environmental Law | 3 | Law | Optimistic | Descriptive |
| | Laubach | 2016 | Epigenetics and Toxic Torts | 3 | Law | Optimistic | Descriptive |
| | Gunther and Felthous | 2015 | Epigenetics and the Law\* | ­ | Law | Precautionary | Reflexive |
| | Tamatea | 2015 | ‘Biologizing’ Psychopathy | 2, 3 | Law | Precautionary | Reflexive |
| | Rothstein | 2013 | Epigenetic Exceptionalism | 3, 7 | Law | Precautionary | Dialectical |
| | Turker | 2012 | Banning Bisphenol A in the United States and Canada | 4 | Law | Optimistic | Instrumental |
| | Khan | 2010 | Preserving Human Potential as Freedom | 3 | Law | Optimistic | Dialectical |
| | Wiener | 2010 | Transgenerational Tort Liability for Epigenetic Diseases | 4 | Law | Optimistic | Descriptive |
| | | | | | | | |
| 7. Discrimination | Saulnier and Dupras | 2017 | Race in the Postgenomic Era\* | 1 | Bioethics | Precautionary | Instrumental |
| | Meloni | 2017 | Race in an Epigenetic Time | 2 | Sociology | Precautionary | Reflexive |
| | Graham et al. | 2016 | Epigenetics and Russia | 9 | Philosophy | Precautionary | Reflexive |
| | Mansfield and Guthman | 2015 | Epigenetic Life | 1, 4 | Geography | Precautionary | Reflexive |
| | Katz | 2013 | The Biological Inferiority of the Undeserving Poor | ­ | Social Work | Precautionary | Reflexive |
| | Sullivan | 2013 | Inheriting Racist Disparities in Health | ­ | Philosophy | Optimistic | Instrumental |
| | Guthman | 2012 | Doing Justice to Bodies? | 2, 5 | Geography | Optimistic | Reflexive |
| | Mansfield and Guthman | 2012 | Race and the New Epigenetic Biopolitics of Environmental Health | 2, 3 | STS | Precautionary | Reflexive |
| | | | | | | | |
| 8. Privacy Protection | Joly et al. | 2016 | Are Data Sharing and Privacy Protection Mutually Exclusive?\* | ­ | Biology | Precautionary | Dialectical |
| | Backes | 2016 | Privacy in Epigenetics\* | 7 | Biology | Neutral | Descriptive (Emp) |
| | Dyke et al. | 2015 | Epigenome Data Release | 7 | Genomics | Precautionary | Descriptive (Emp) |
| | Erwin | 2015 | Ethical Issues in Epigenetic Testing | 7 | Law | Precautionary | Descriptive |
| | Joly et al. | 2015 | Risk of Re-identification of Epigenetic Methylation Data\* | ­ | Epigenomics | Precautionary | Dialectical |
| | Thomas | 2015 | Epigenetics and Child Psychiatry | 6, 7 | Law | Precautionary | Descriptive |
| | Terry | 2015 | Developments in Genetic and Epigenetic Data Protection… | ­ | Law | Precautionary | Dialectical |
| | Philibert et al. | 2014 | Methylation Array Data Can Simultaneously Identify Individuals and … | ­ | Epigenomics | Precautionary | Descriptive (Emp) |

## Slide 3
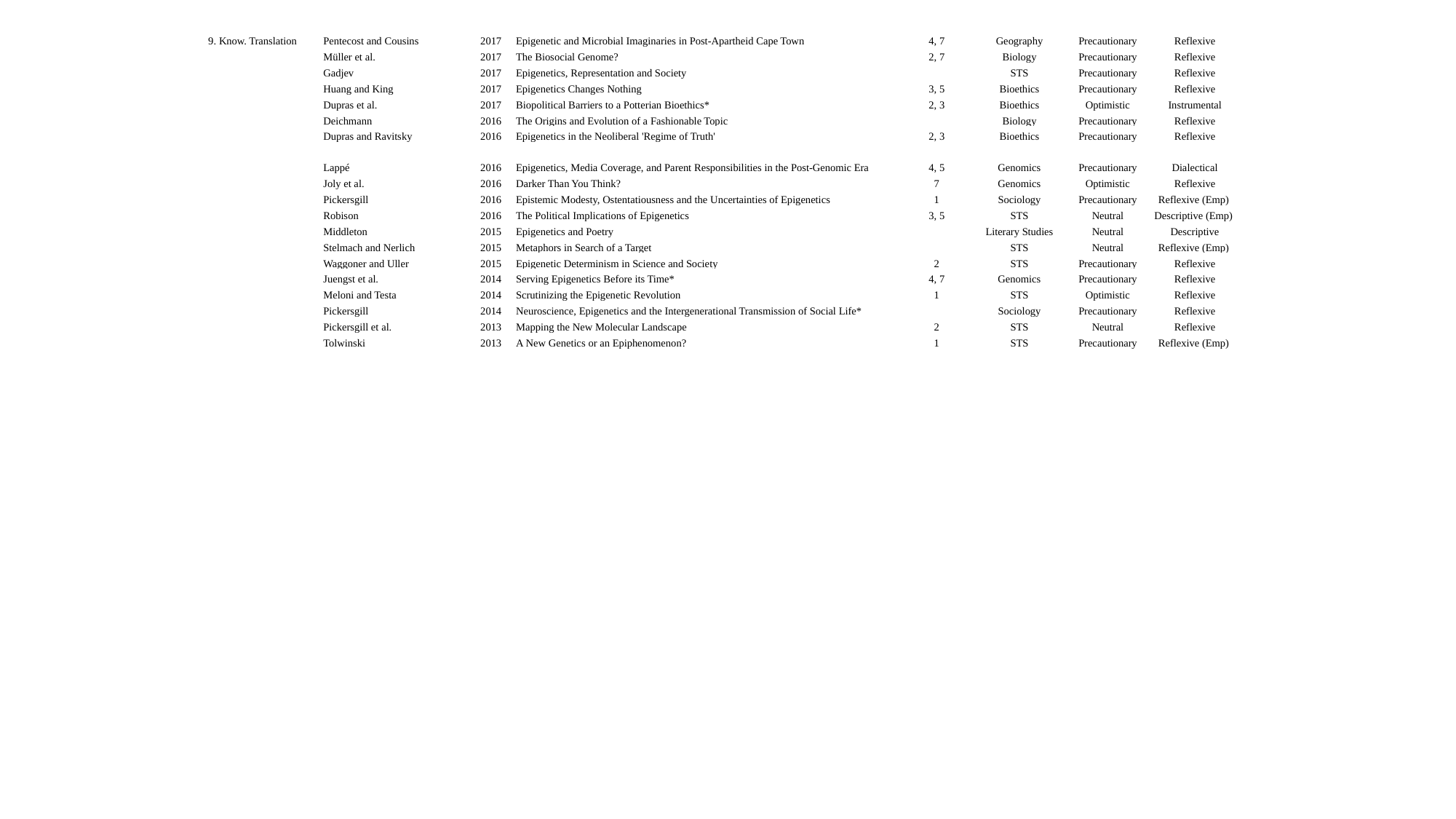

| 9. Know. Translation | Pentecost and Cousins | 2017 | Epigenetic and Microbial Imaginaries in Post-Apartheid Cape Town | 4, 7 | Geography | Precautionary | Reflexive |
| --- | --- | --- | --- | --- | --- | --- | --- |
| | Müller et al. | 2017 | The Biosocial Genome? | 2, 7 | Biology | Precautionary | Reflexive |
| | Gadjev | 2017 | Epigenetics, Representation and Society | ­ | STS | Precautionary | Reflexive |
| | Huang and King | 2017 | Epigenetics Changes Nothing | 3, 5 | Bioethics | Precautionary | Reflexive |
| | Dupras et al. | 2017 | Biopolitical Barriers to a Potterian Bioethics\* | 2, 3 | Bioethics | Optimistic | Instrumental |
| | Deichmann | 2016 | The Origins and Evolution of a Fashionable Topic | ­ | Biology | Precautionary | Reflexive |
| | Dupras and Ravitsky | 2016 | Epigenetics in the Neoliberal 'Regime of Truth' | 2, 3 | Bioethics | Precautionary | Reflexive |
| | Lappé | 2016 | Epigenetics, Media Coverage, and Parent Responsibilities in the Post-Genomic Era | 4, 5 | Genomics | Precautionary | Dialectical |
| | Joly et al. | 2016 | Darker Than You Think? | 7 | Genomics | Optimistic | Reflexive |
| | Pickersgill | 2016 | Epistemic Modesty, Ostentatiousness and the Uncertainties of Epigenetics | 1 | Sociology | Precautionary | Reflexive (Emp) |
| | Robison | 2016 | The Political Implications of Epigenetics | 3, 5 | STS | Neutral | Descriptive (Emp) |
| | Middleton | 2015 | Epigenetics and Poetry | ­ | Literary Studies | Neutral | Descriptive |
| | Stelmach and Nerlich | 2015 | Metaphors in Search of a Target | ­ | STS | Neutral | Reflexive (Emp) |
| | Waggoner and Uller | 2015 | Epigenetic Determinism in Science and Society | 2 | STS | Precautionary | Reflexive |
| | Juengst et al. | 2014 | Serving Epigenetics Before its Time\* | 4, 7 | Genomics | Precautionary | Reflexive |
| | Meloni and Testa | 2014 | Scrutinizing the Epigenetic Revolution | 1 | STS | Optimistic | Reflexive |
| | Pickersgill | 2014 | Neuroscience, Epigenetics and the Intergenerational Transmission of Social Life\* | ­ | Sociology | Precautionary | Reflexive |
| | Pickersgill et al. | 2013 | Mapping the New Molecular Landscape | 2 | STS | Neutral | Reflexive |
| | Tolwinski | 2013 | A New Genetics or an Epiphenomenon? | 1 | STS | Precautionary | Reflexive (Emp) |
